# PI3Kδ inhibitor idelalisib in combination with BTK inhibitor ONO/GS-4059 in diffuse large B cell lymphoma with acquired resistance to PI3Kδ and BTK inhibitors

**DOI:** 10.1371/journal.pone.0171221

**Published:** 2017-02-08

**Authors:** Anella Yahiaoui, Sarah A. Meadows, Rick A. Sorensen, Zhi-Hua Cui, Kathleen S. Keegan, Robert Brockett, Guang Chen, Christophe Quéva, Li Li, Stacey L. Tannheimer

**Affiliations:** Gilead Sciences, Inc., Foster City, California, United States of America; Universidad de Navarra, SPAIN

## Abstract

Activated B-cell-like diffuse large B-cell lymphoma relies on B-cell receptor signaling to drive proliferation and survival. Downstream of the B-cell receptor, the key signaling kinases Bruton’s tyrosine kinase and phosphoinositide 3-kinase δ offer opportunities for therapeutic intervention by agents such as ibrutinib, ONO/GS-4059, and idelalisib. Combination therapy with such targeted agents could provide enhanced efficacy due to complimentary mechanisms of action. In this study, we describe both the additive interaction of and resistance mechanisms to idelalisib and ONO/GS-4059 in a model of activated B-cell-like diffuse large B-cell lymphoma. Significant tumor regression was observed with a combination of PI3Kδ and Bruton’s tyrosine kinase inhibitors in the mouse TMD8 xenograft. Acquired resistance to idelalisib in the TMD8 cell line occurred by loss of phosphatase and tensin homolog and phosphoinositide 3-kinase pathway upregulation, but not by mutation of *PIK3CD*. Sensitivity to idelalisib could be restored by combining idelalisib and ONO/GS-4059. Further evaluation of targeted inhibitors revealed that the combination of idelalisib and the phosphoinositide-dependent kinase-1 inhibitor GSK2334470 or the AKT inhibitor MK-2206 could partially overcome resistance. Characterization of acquired Bruton’s tyrosine kinase inhibitor resistance revealed a novel tumor necrosis factor alpha induced protein 3 mutation (*TNFAIP3* Q143*), which led to a loss of A20 protein, and increased p-IκBα. The combination of idelalisib and ONO/GS-4059 partially restored sensitivity in this resistant line. Additionally, a mutation in Bruton’s tyrosine kinase at C481F was identified as a mechanism of resistance. The combination activity observed with idelalisib and ONO/GS-4059, taken together with the ability to overcome resistance, could lead to a new therapeutic option in activated B-cell-like diffuse large B-cell lymphoma. A clinical trial is currently underway to evaluate the combination of idelalisib and ONO/GS-4059 (NCT02457598).

## Introduction

B-cell receptor (BCR) signaling is a key driver of pathogenesis in many types of lymphoid malignancies, including chronic lymphocytic leukemia (CLL) and activated B-cell-like diffuse large B-cell lymphoma (ABC DLBCL) [1]. The BCR complex consists of an immunoglobulin that is non-covalently coupled to its CD79A (Ig-A)/ CD79B (Ig-B) subunits. Antigen binding leads to CD79A and CD79B immunoreceptor tyrosine-based activation motifs phosphorylation by spleen tyrosine kinase (SYK) and Lyn or other SRC family kinase (SFK) members. This initiates a signaling cascade that consequently activates phosphoinositide 3-kinase (PI3K), Bruton’s tyrosine kinase (BTK), and other downstream signaling pathways, including activation of NF-κB [2, 3].

The class I PI3K family, which includes the catalytic p110 α, β, γ and δ isoforms, are often mutationally or constitutively activated in a variety of cancers [4]. PI3Kδ expression is restricted to leukocytes, and is physiologically the predominant isoform in B cells. PI3Kδ has also been demonstrated to play an active role in driving B cell malignancies such as CLL and B-NHL [5, 6].

Clinical trials have recently demonstrated significant efficacy with inhibitors that disrupt BCR signaling, including Zydelig^®^ (idelalisib) and Imbruvica^®^ (ibrutinib) [7, 8]. Idelalisib is a first-in-class, selective inhibitor of PI3Kδ approved for the treatment of relapsed/refractory CLL (in combination with rituximab), follicular lymphoma, and small lymphocytic lymphoma [9]. Ibrutinib is a BTK inhibitor approved for treatment of CLL, mantle cell lymphoma and Waldenström's macroglobulinemia. While neither agent is currently approved for ABC DLBCL, ongoing trials are evaluating the potential of agents that target downstream signaling proteins such as PI3Kδ, BTK, and SYK that are predicted to impact survival and proliferation pathways in ABC DLBCL. One such agent, the selective and potent BTK inhibitor ONO/GS-4059, reported 35% overall response rate in relapsed/refractory non-germinal center B-cell DLBCL [10]. Despite the efficacy of these targeted agents in DLBCL, the low response rates, short duration of response and potential for acquired resistance highlights the necessity for combination therapy. In this study, we set out to characterize the potential antitumor activity of combining idelalisib with ONO/GS-4059, as well as to define the mechanisms of resistance for each class of agent in a model of ABC DLBCL.

## Materials and methods

### Cell lines and compound reagents

TMD8 cells were obtained from the Tokyo Medical and Dental University, and OCI-LY10 cells were obtained from University Health Network. Both cell lines were cultured in RPMI-1640 medium supplemented with 20% FBS, 100 U/mL penicillin, and 100 μg/mL streptomycin (Life Technologies, Carlsbad, CA). Idelalisib and ibrutinib resistant TMD8 were cultured in the presence of idelalisib (1 μM) or ibrutinib (10–20 nM), respectively, and grown in a humidified atmosphere of 5% CO_2_ and 95% air at 37°C. Compounds used in this study include: idelalisib (Gilead Sciences, Inc., Foster City, CA), GS-649443 (Gilead Sciences) [11], BYL719, AZD-6482, GDC-0941, MK-2206 and GSK2334470 (Selleckchem, Houston, TX) [12–16], ibrutinib (Shanghai Medicilon Inc., Shanghai, China), and ONO/GS-4059 (Ono Pharmaceutical Co., Trenton, NJ).

### Generation of idelalisib and ibrutinib resistant TMD8 cells

Idelalisib-resistant TMD8 cells were generated by continuous passage in the presence of 1 μM idelalisib for 8 weeks until stable resistance to idelalisib was established (TMD8^IDELA-R^). Ibrutinib-resistant TMD8 cells were generated by continuous passaging in the presence of ibrutinib for 12 weeks then dose-escalating to 10 or 20 nM until stable resistance to ibrutinib was established (TMD8^BTKi-R^). Parallel cultures were grown in the presence of 0.1% DMSO as passage-matched, drug-sensitive control lines (TMD8^IDELA-S^ and TMD8^BTKi-S^). Sensitive and resistant TMD8 cells were clonally isolated through two rounds of single cell limiting dilution.

### Cell viability and apoptosis assays

Cells were seeded at 20,000 cells/well in 96-well plates. Test compounds were added in quadruplicate using the HP D300 Digital Dispenser (Hewlett Packard Inc.). Inhibition of cell viability was assessed after 96 hours using CellTiter Glo^®^ Luminescent Cell Viability assay (Promega Corp., Madison, WI). Data was plotted as percent of vehicle (DMSO) control using GraphPad Prism (San Diego, CA). For apoptosis assays, cells were seeded at 0.2 X 10^6^/mL in a 12 well dish. Apoptosis was measured after 48 hours using FITC Annexin V Apoptosis Detection Kit II or PE Annexin V Apoptosis Detection Kit I (BD Biosciences, San Jose, CA) following manufacturer's protocol. Labeled cells were measured by flow cytometry using BD LSRII and analyzed using FACSDiva^™^ (BD Biosciences).

### Immunoblotting

Cells were grown overnight in RPMI-1640 medium supplemented with 10% FBS and treated the following morning with idelalisib (420nM), ONO/GS-4059 (320nM), or in combination (protein-adjusted C_max_ values), or idelalisib (1μM), MK2206 (1μM), GSK2334470 (1μM) for 2 hours or 24 hours. Cells were harvested in lysis buffer (Cell Signaling Technology) containing: Protease Inhibitor Cocktail (Roche Diagnostics Corp), and phosphatase inhibitor sets 1 and 2 (EMD Millipore). Following 30 minutes on ice, cell lysates were cleared by centrifugation at 12,500 rpm for 10 minutes at 4°C. Lysates were analyzed by Simple Western using Peggy Sue^™^ (ProteinSimple, San Jose, CA; referred to in the text as Simple Western) or by Novex SDS-PAGE gels (Invitrogen, Carlsbad, CA) blotted to Immobilon-F membrane (EMD Millipore, Billerica, MA). A standard curve using recombinant proteins was generated to measure PI3K isoform concentration by Simple Western; data was processed using Compass software (ProteinSimple). Western blots were quantified using LI-COR Imager and LI-COR Odyssey Image Studio Software version 5.2 (LI-COR Biosciences, Lincoln, NE). The following antibodies were purchased from Cell Signaling Technology (Danvers, MA): p-AKT (S473) (#4058 rabbit monoclonal for Simple Western; #9271 rabbit polyclonal for western blots), p-AKT (T308) #2965 rabbit monoclonal, AKT (#2920 mouse monoclonal), p-ERK (T202/Y204) (#9101 rabbit polyclonal for Simple Western, #9106 mouse monoclonal for western blots), ERK (#9102 rabbit polyclonal), p-S6RP (S235/236) (#4856 rabbit monoclonal for Simple Western), S6 (#2317 mouse monoclonal), p-GSK3β (S9) (#9336 rabbit polyclonal),p-STAT3 (Y705) (#9131 rabbit polyclonal), p-SFK (Y416) (#6943 rabbit monoclonal), p-IκBα (S32) (#2859 rabbit monoclonal), IκBα (#2814 mouse monoclonal), p-SYK (Y525/526) (#2710 rabbit monoclonal), p-BTK (Y223) (#5082 rabbit polyclonal), BTK (#8547 rabbit monoclonal), PTEN (#9559 rabbit monoclonal), A20/TNFAIP3 (#5630 rabbit monoclonal), and actin (#4968 rabbit polyclonal). The c-MYC antibody (#ab32072 rabbit monoclonal) was purchased from Abcam, Inc. (Cambridge, MA). GSK3β (#05–412 mouse monoclonal) and the actin used in western blots (#MAB1501 mouse monoclonal) were purchased from EMDMillipore. PI3Kγ (#ABD-026 mouse monoclonal) was purchased from Jena Bioscience (Jena, Germany). The p-SYK (Y352) antibody (#SAB4301384 rabbit polyclonal) was purchased from Sigma-Aldrich (St. Louis, MO). Secondary IRDye-conjugated anti-mouse and anti-rabbit antibodies were purchased from LI-COR Biosciences.

### *In vivo* treatment study

These studies were approved by the Institutional Animal Care and Use Committee at Omeros Corp. in Seattle, Washington, which complies with all provisions of the United States Public Health Service Policy on Humane Care and Use of Laboratory Animals (NIH Publication No. 15–8013) and the United States Department of Agriculture Animal Welfare Regulations (United States Code of Federal Regulations, 9 C.F.R. Parts 1–4). Animal care and research procedures were performed in dedicated rooms within their animal facility using their program of husbandry under the direction of a veterinarian board certified by the American College of Laboratory Animal Medicine. The animal facility provides a barrier-type environment free of all rodent pathogens using individually ventilated microisolator cages for mice; irradiated corncob bedding, ad libitum irradiated food (PicoLab Rodent Diet, St. Louis, MO), ad libitum ultrafiltered and UV irradiated water, and species-appropriate nesting material as enrichment. Room temperatures were maintained at 70–72 degrees F and 30–70% relative humidity with a 12:12 light: dark photoperiod. Male CB17-SCID mice were treated with 1.44 Gy whole body irradiation using a ^60^Co radiation source. After 72 hours, 1×10^7^ TMD8 cells were inoculated subcutaneously into the right flank. When tumors reached a mean volume of 200mm^3^, mice were randomly assigned to groups (n = 13) using Vivo Manager^®^ software (Biosystemes, Couternon, France). Mice were administered twice daily either vehicle, GS-649443 at 1 and 5 mg/kg, or ONO/GS-4059 at 5 and 10 mg/kg, alone or in combination, by oral gavage at a dosing volume of 5 ml/kg. All test compounds were formulated into 5% (v/v) N-Methyl-2-pyrrolidone (NMP) / 55% (v/v) Polyethylene Glycol 300 (PEG) 300 / 40% (v/v) Water / 1% (w/v) Vitamin E D-α-Tocopherol Polyethylene Glycol 1000 Succinate (TPGS). The tumor volume was calculated, taking length to be the longest diameter across the tumor and width to be the corresponding perpendicular diameter, using the following formula: (length×width^2^)/2. The tumor growth inhibition (TGI) was calculated based on tumor volume (TV) measured at the beginning and the end of compound or vehicle treatment:

$$TGI=1-(\frac{TV\;end\;of\;compound-TV\;begining\;of\;compound}{TV\;end\;of\;vehicle-TV\;begining\;of\;vehicle})\times 100$$

### Immunohistochemistry

Immunohistochemistry was performed on the Ventana Discovery Ultra (Ventana Medical Systems, Tucson, AZ) autostainer using the manufacturer’s instructions and reagents. The slides were immunolabeled for p-S6 (S235/236) (Cat #4858, Cell Signaling Technology) and c-MYC (Cat #ab32072, Abcam Inc.), and counterstained with hematoxylin.

### Genomic profiling

Characterization of mutations and gene expression were identified by whole exome sequencing (WES; Genewiz, Inc., South Plainfield, NJ) and RNA-Seq (Expression Analysis, Inc, Morrisville, NC), respectively. DNA sequencing reads were aligned to human reference genome by BWA [17] and single nucleotide variants were identified using VarScan version 2.3 [18] and were annotated using SnpEff version 4.0 [19]. Putative somatic mutations were prioritized by mutant allele frequency, recurrence and predicted functional impact. RNA sequencing reads were aligned to human reference genome by STAR [20] and RNA abundance was quantified using RSEM [21]. The Bioconductor package edgeR [22] was used to normalize sequence count and limma (Bioconductor [23]) was used to conduct differential gene expression analysis.

### Statistical analysis

Cell viability EC_50_ was determined using a sigmoidal dose-response (variable slope) curve generated from quadruplicate samples. Statistical significance was performed by student’s t-test for cell viability and two-tailed paired t-test for apoptosis experiments (GraphPad). The mixed effect Analysis of Variance (ANOVA) model for repeated measure was used to determine the treatment effect on tumor growth. The model fitted on tumor volume included factors of treatment, time, and their interaction. Baseline tumor volume was also included as covariate. The covariance among repeated measurements was assumed with ante-dependence structure. The 8 mono- and combination-treatment groups were compared to vehicle control with Dunnett’s multiple comparison adjustment. Each of the 4 combination-treatment groups was also compared to the 2-corresponding mono-dose groups. A multivariate t method was applied for multiple comparison adjustment. A logarithm transformation was applied on tumor volume to meet model assumptions. The analysis was performed using SAS^®^ v9.2 (SAS Institute, Inc., Cary, NC). A one-way ANOVA method was used to compare treatment groups to vehicle control in pharmacodynamic tumor samples. Log transformation was applied when needed. No multiple comparison adjustments were applied.

## Results

### Idelalisib and ONO/GS-4059 additively affect cell viability, apoptosis, and key survival and proliferation pathways in DLBCL

**Fig 1:**
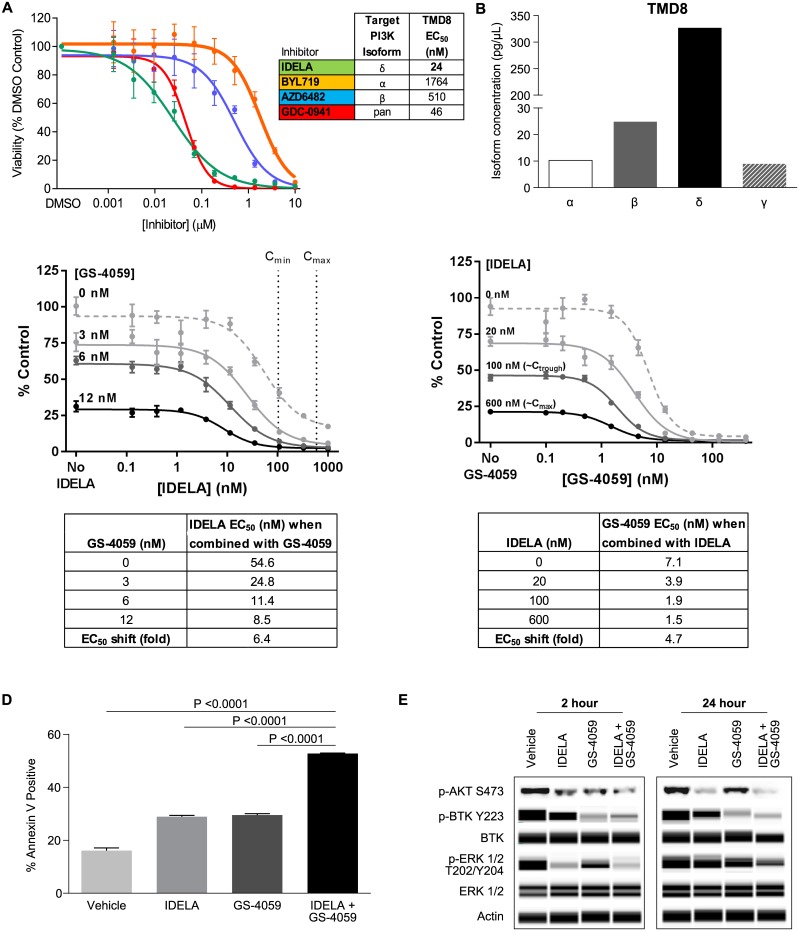
TMD8 as a model for evaluation of PI3Kδ and BTK inhibition. (A) Cell viability with isoform-specific PI3K inhibitors was assessed by CellTiter Glo after 96 hours, mean ± SEM, n = 4. (B) PI3K isoform expression in TMD8 cells using Simple Western, concentration (pg/μL) quantitated by recombinant protein standards, representative experiment shown of n = 3. (C) Cell viability of TMD8 was assessed with idelalisib in combination with ONO/GS-4059 at fixed concentrations (3, 6, or 12 nM) or with ONO/GS-4059 in combination with idelalisib at fixed concentrations (20, 100 or 600 nM) after 96 hours, mean ± SEM, n = 8. (D) Cells were treated with idelalisib (420 nM), ONO/GS-4059 (320 nM) or in combination for 48 hours and apoptosis was assessed by FITC Annexin V staining, and measured by flow cytometry, mean values ± SD, n = 4. (E) Cells were treated with idelalisib (420 nM), ONO/GS-4059 (320 nM) or in combination for 2 and 24 hours; lysates were analyzed by western blot (p-AKT S473) and Simple Western, representative experiment shown of n = 3.

A panel of 28 DLBCL cell lines were screened for their sensitivity to idelalisib and ONO/GS-4059 as assessed by changes in cell viability (S1 Table). Three ABC DLBCL cell lines, TMD8, OCI-LY10, and Ri-1, were identified as being sensitive to both agents. Cell viability assessment using isoform-specific inhibitors showed that the TMD8 cell line appeared to be primarily driven by the PI3Kδ isoform. The EC_50_ for the PI3Kδ-selective inhibitor idelalisib and the pan PI3K inhibitor GDC-0941, was 24 nM and 46 nM, respectively (Fig 1A). TMD8 was relatively insensitive to the potent and selective PI3Kα- and β-selective inhibitors BYL719 and AZD-6482, with EC_50_ values of 1764 nM and 510 nM, respectively. Furthermore, isoform protein expression analysis revealed that the catalytic subunit of p110δ was most highly expressed in TMD8 cells (327 pg/μL) as compared to p110α, p110β, and p110γ (10, 25, and 9 pg/μL, respectively) (Fig 1B). Both TMD8 and OCI-LY10 are BCR-dependent lines that exhibit chronic activated B-cell signaling due to mutations in *CD79A/CD79B* and *MYD88* [24]. These lines were chosen to evaluate the effects of idelalisib and ONO/GS-4059 in combination. The idelalisib concentrations experimentally evaluated covered a clinically relevant range, and showed an additive effect in combination with ONO/GS-4059 on cell viability in TMD8 and OCI-LY10. The addition of ONO/GS-4059 (3, 6, and 12 nM) to idelalisib in the TMD8 line shifted the EC_50_ from 55 nM to 25, 11, and 9 nM, respectively (Fig 1C). When idelalisib was added in combination at concentrations modeling clinical C_min_ and C_max_ (103 and 591 nM, respectively), ONO/GS-4059 EC_50_ shifted from 7 nM (single agent) to 2 and 1.5 nM. In OCI-LY10, the addition of ONO/GS-4059 (25 nM) maximally shifted the EC_50_ of idelalisib from 122 to 13 nM (S1 Fig). In TMD8, the combination of idelalisib and ONO/GS-4059 induced 53% apoptosis as measured by annexin V staining, more than either single agent alone (idelalisib = 29%, ONO/GS-4059 = 30%) (Fig 1D).

The effect of the combination of these compounds was characterized on downstream signaling pathways implicated in malignant B-cell survival and proliferation. Idelalisib elicited a stronger inhibition of p-AKT (S473) and p-ERK (T202/Y204) (58% and 71%, respectively) than ONO/GS-4059 at 2 hours (46% and 48%, respectively) (Fig 1E). As expected, ONO/GS-4059 strongly inhibited BTK activation as measured by p-BTK (Y223) (59%). Evaluation of the combination of idelalisib and ONO/GS-4059 did not show an effect greater than observed with either agent alone. At an extended time point (24 hours), a sustained effect of both agents in combination was seen on p-AKT (S473), p-BTK (Y223) and p-ERK (T202/Y204) (83%, 66% and 36% inhibition respectively, as compared to DMSO control). When comparing inhibition of the combination versus the most effective single agent, the percent inhibition was 28%, 22% and 34% for p-AKT, p-BTK and p-ERK, respectively. These data are in line with the observed additive effect on cell viability and apoptosis with the combination of idelalisib and ONO/GS-4059.

### Inhibition of PI3Kδ and BTK leads to TMD8 tumor regression

**Fig 2:**
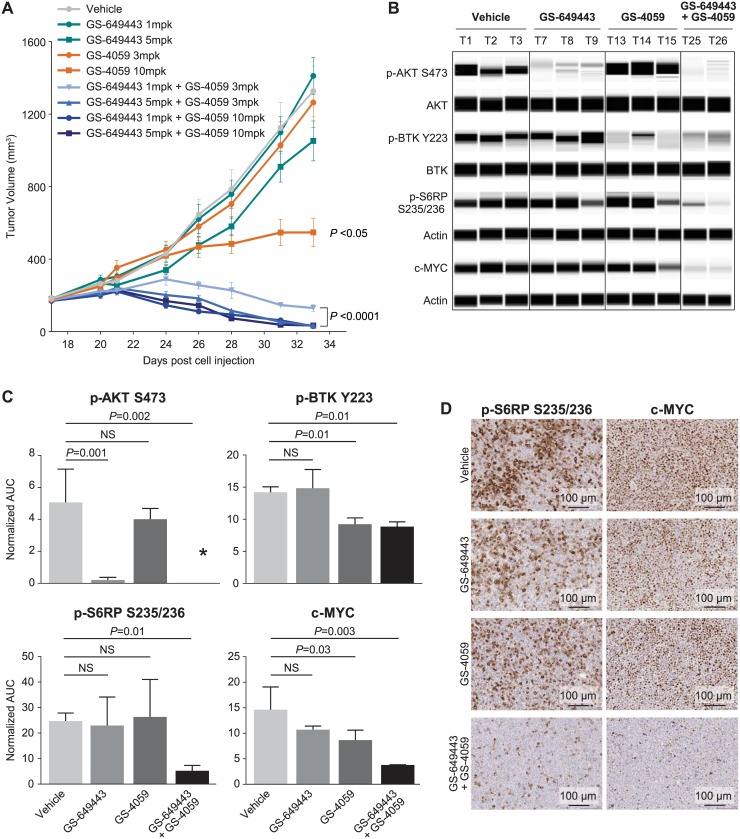
In vivo inhibition of PI3Kδ and BTK leads to TMD8 tumor regression. (A) Response of randomized TMD8 xenograft tumors (n = 13 per group) treated with PI3Kδ inhibitor (GS-649443, 1 or 5 mg/kg, BID), BTK inhibitor (ONO/GS-4059, 3 or 10 mg/kg, BID) or combination. Tumor volumes are expressed as mean ± SEM with P<0.05, P <0.0001 as compared to vehicle animals. (B) Tumors from vehicle, GS-649443 (5 mg/kg), ONO/GS-4059 (10 mg/kg) or GS-649443 + ONO/GS-4059 (5 mg/kg + 10 mg/kg) were collected 2 hours post morning dose on day 3 of dosing, ground and lysed. Protein expression was analyzed using Simple Western; T = tumor. (C) Average of same tumors (from (B)) for each treatment group (n = 3 for vehicle, GS-649443 and ONO/GS-4059 groups; n = 2 for combination); proteins were quantitated by AUC, p-BTK Y223 was normalized to total BTK protein, p-S6RP S235/236 and c-MYC were normalized to actin, and p-AKT S473 was normalized to total AKT, mean ± SD. Asterisk indicates that the AUC combination result is 0. (D) p-S6RP S235/236 and c-MYC expression detected by IHC in tumors from vehicle, GS-649443 (5 mg/kg), ONO/GS-4059 (10 mg/kg) or GS-649443 + ONO/GS-4059 (5 mg/kg + 10 mg/kg) treatment groups. Images were taken at 20X magnification.

To extend the observed additivity seen *in vitro*, the efficacy of PI3Kδ and BTK inhibition in combination was assessed in a TMD8 xenograft model. Due to the short half-life of idelalisib in mice, the PI3Kδ tool compound GS-649443 was used *in vivo*. GS-649443 is more potent but displays similar selectivity to idelalisib across the class I PI3K family (IC_50_ = 1160, 419, 0.3, 24 nM for PI3Kα, PI3Kβ, PI3Kδ and PI3Kγ, respectively; S1 Table, S2 Fig) [11, 25]. Suboptimal doses of GS-649443 were chosen for our xenograft evaluation to allow a window for observation of additivity of PI3Kδ and BTK inhibition. Evaluation of tumor volumes showed that GS-649443 as a single agent (1 or 5 mg/kg, BID) did not inhibit TMD8 tumor growth (Fig 2A). Similarly, ONO/GS-4059 at 3 mg/kg BID did not lead to a significant inhibition of tumor growth. In contrast, a 75% decrease in tumor volume was observed with the highest dose of ONO/GS-4059 (10 mg/kg BID, *P* < 0.05). The combination of GS-649443 and ONO/GS-4059 at the lowest and highest dose groups led to a 110% and 120% inhibition of tumor growth, respectively. All combinations of GS-649443 and ONO/GS-4059 strongly inhibited tumor growth, leading to tumor regression (*P* < 0.0001). No decreases in body weight were observed in any of the treatment groups (data not shown). Pharmacodynamic results in the individual TMD8 mouse tumors showed a 35% decrease in p-BTK with ONO/GS-4059 (Fig 2B and 2C). While GS-649443 and ONO/GS-4059 as single agents had no effect on p-S6RP, a 79% decrease was observed in combination. This effect was confirmed by immunohistochemical (IHC) analysis of p-S6RP (Fig 2D). Additionally, IHC and Simple Western analysis of c-MYC on these same tumor samples revealed that the combination of GS-649443 and ONO/GS-4059 showed a dramatic decrease in c-MYC whereas single agent treatment showed little to no effect. c-MYC is a well characterized proto-oncogene which has been correlated with poor clinical prognosis and drug resistance. These data demonstrate that inhibition of PI3Kδ and BTK signaling pathways significantly impacts downstream signaling pathways as measured by p-S6RP and c-MYC, translating into additive antitumor efficacy in the TMD8 model of ABC DLBCL.

### Acquired idelalisib resistance is overcome by the combination of idelalisib and ONO/GS-4059

**Fig 3:**
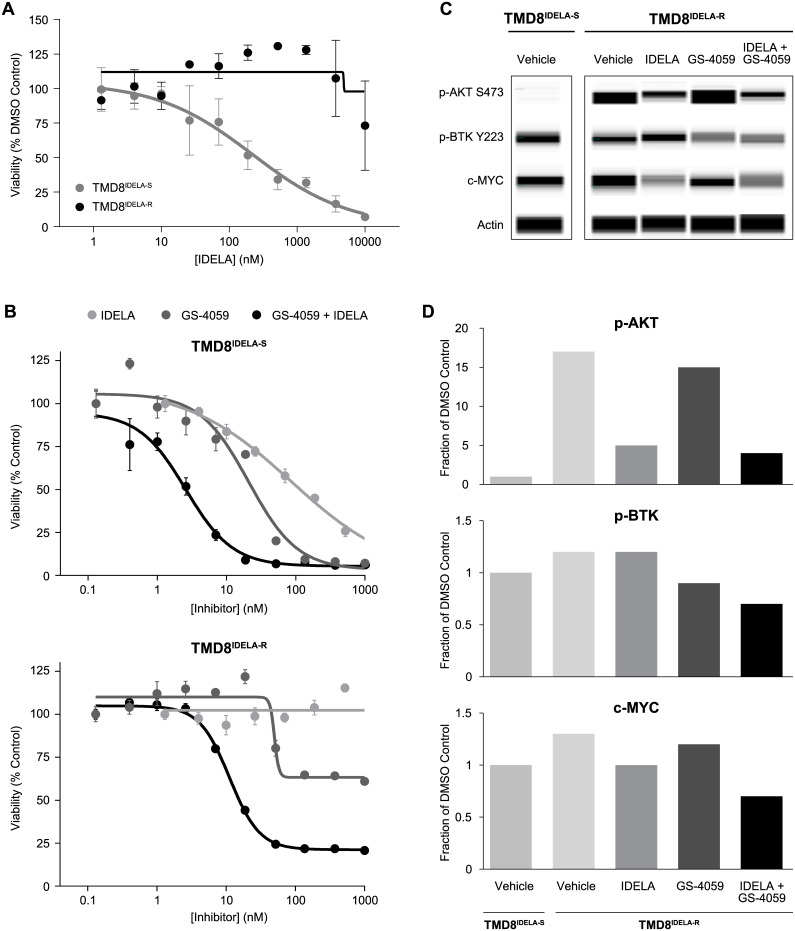
Combination of idelalisib and ONO/GS-4059 can overcome TMD8 acquired idelalisib resistance. (A) Cell viability of vehicle control line (TMD8^IDELA-S^) or idelalisib resistant line (TMD8^IDELA-R^) in response to idelalisib treatment, 96 hour CellTiterGlo assay, mean ± SEM, n = 4. (B) Cell viability of TMD8^IDELA-S^ (top graph) or TMD8^IDELA-R^ cells (bottom graph) treated with idelalisib, ONO/GS-4059 or ONO/GS-4059 in combination with idelalisib (1 μM), n = 4, mean ± SEM. Combination curve is normalized to the single agent alone. (C) Protein lysates from TMD8^IDELA-S^ (vehicle treated) and TMD8^IDELA-R^ treated with vehicle, idelalisib (420 nM), ONO/GS-4059 (320 nM) or in combination for 2 hours were generated and analyzed in a single run using Simple Western, representative image of a single run of n = 3. (D) Quantification of Fig 3C showing AUC normalization to actin, and values normalized to TMD8^IDELA-S^ DMSO treated control for all treatment groups from a single run.

To understand the resistance to targeted agents observed clinically, we created an *in vitro* model of acquired idelalisib resistance in the TMD8 cell line. Long-term idelalisib treatment (1 μM) led to the establishment of a cell line with acquired resistance (TMD8^IDELA-R^), while a passage-matched, vehicle control line (TMD8^IDELA-S^) remained sensitive to idelalisib as assessed by cell viability (EC_50_ > 10 μM, EC_50_ = 220 nM, respectively) (Fig 3A). Acquired resistance was not due to the presence of a subpopulation of innately resistant cells, as the evaluation of 8 single cell clonal isolates all showed resistance to idelalisib, compared to TMD8^IDELA-S^ (S3 Fig). Additionally, RNAseq analysis of the multidrug resistance (MDR) family of ATP-binding cassette transporters in TMD8^IDELA-S^ as compared to TMD8^IDELA-R^ indicated that upregulation of MDR was not a likely mechanism of resistance (S4 Fig).

In order to determine if ONO/GS-4059 could overcome resistance in the TMD8^IDELA-R^ line, single agent and combination activity was assessed using clinically relevant doses. As expected, both idelalisib and ONO/GS-4059 showed activity on the TMD8^IDELA-S^ line, with additive inhibition seen in combination (Fig 3B, top graph). However, the TMD8^IDELA-R^ line showed resistance to both idelalisib and ONO/GS-4059 (Fig 3B, bottom graph). Sensitivity was almost completely restored when both agents were used in combination, shifting the ONO/GS-4059 EC_50_ to 11 nM in the presence of 1 μM idelalisib. Measurement of downstream cell signaling proteins (p-AKT, p-BTK, and c-MYC) in the TMD8^IDELA-R^ line showed higher constitutive p-AKT levels (16-fold), with little to no upregulation of p-BTK and c-MYC (Fig 3C and 3D). Idelalisib treatment, but not ONO/GS-4059, inhibited p-AKT with no greater inhibition observed in combination. ONO/GS-4059 had limited activity on inhibition of p-BTK, especially when compared previously to the TMD8 parental line (Fig 1E). Idelalisib showed modest c-MYC inhibition, but a stronger downmodulation was observed in combination. Taken together this suggests that only combination treatment can overcome the signaling dysregulation affecting multiple pathways.

### Profiling of TMD8 idelalisib-resistant cell line shows PTEN loss and PI3K pathway upregulation

**Fig 4:**
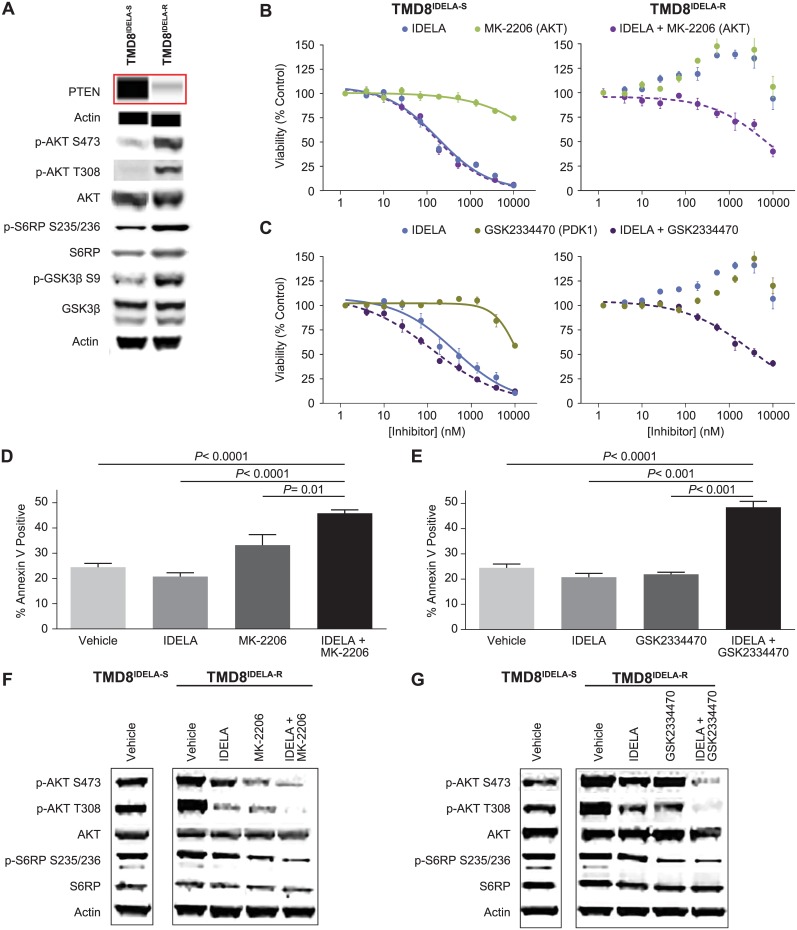
PI3K upregulation in acquired resistance is sensitive to idelalisib and AKT or PDK1 inhibitor combinations. (A) Protein lysates were generated from TMD8^IDELA-S^ and TMD8^IDELA-R^ cells, and analyzed by western blot (p-AKT S473, p-AKT T308, AKT, p-S6RP S235/236, S6RP, p-GSK3β S9, GSK3β) and Simple Western (PTEN). (B) Cell viability of TMD8^IDELA-S^ (left) and TMD8^IDELA-R^ (right) cells treated with idelalisib, MK-2206 or combination of idelalisib and MK-2206 (1 μM), 96 hours CellTiterGlo assay, mean ± SEM, n = 4. Combination curve is normalized to the single agent alone. (C) Cell viability of TMD8^IDELA-S^ (left) and TMD8^IDELA-R^ (right) cells treated with idelalisib, GSK2334470 or combination of idelalisib and GSK2334470 (3 μM), 96 hours CellTiterGlo assay, mean ± SEM, n = 4. Combination curve is normalized to the single agent alone. (D) TMD8^IDELA-R^ cells were treated with vehicle, idelalisib (1 μM), MK-2206 (1 μM), and idelalisib plus MK-2206, or (E) Vehicle, idelalisib (1 μM), GSK2334470 (1 μM), and idelalisib plus GSK2334470 for 48 hours. Apoptosis was assessed by 7AAD and PE annexin V staining, and analyzed by flow cytometry, mean ± SD, n = 3. (F) TMD8^IDELA-R^ cells were treated with vehicle, idelalisib (1 μM), MK-2206 (1 μM), or idelalisib plus MK-2206 and (G) Vehicle, idelalisib (1 μM), GSK2334470 (1 μM), or idelalisib plus GSK2334470 for 2 hours. Protein lysates were generated and analyzed by western blot, representative experiment of n = 3. TMD8^IDELA-S^ vehicle control was included as a reference on the same membrane.

To further characterize the mechanism of resistance to idelalisib, TMD8^IDELA-R^ single cell clones were analyzed by WES, RNAseq, phosphoproteomics and profiling using targeted small molecule inhibitors. No mutations in *PIK3CD* or other PI3K pathway regulators, including *phosphatase and tensin homolog (PTEN)*, were identified by WES as a mechanism of acquired resistance. However, protein profiling of the PI3K pathway revealed PTEN loss in the TMD8^IDELA-R^ which was not predicted by WES or RNAseq (Fig 4A). Additionally, RNAseq analysis of TMD8^IDELA-R^ showed an upregulation of *PIK3CG* mRNA encoding for the catalytic subunit of the class I PI3Kγ isoform, an observation that was confirmed at the protein level (S5A and S5B Fig). Profiling of other PI3K catalytic subunits revealed no other alterations in protein expression (data not shown). TMD8^IDELA-R^ was not sensitive to the PI3Kδ/γ-dual inhibitor duvelisib (IPI-145) suggesting that the upregulation of PI3Kγ may not be contributing to the observed resistance (S5C Fig). This line also showed maintained innate resistance to PI3Kα (BYL719) and PI3Kβ (AZD6482) inhibitors (EC_50_ > 3 μM, data not shown), while the pan-PI3K inhibitor BKM120 showed some decrease in inhibitory activity (EC_50_ 0.76 vs 1.2 uM for DMSO vs. resistant line) as it has selectivity for PI3K α>δ>β>γ [26]. Phosphoprotein characterization was performed to assess the activation state of multiple pathways known to play a role in cell survival and proliferation. This analysis revealed that the PI3K pathway was constitutively upregulated in TMD8^IDELA-R^, as shown by upregulation of p-AKT S473 and T308, p-S6RP S235/236, and p-GSK3β (Fig 4A). Additional signaling nodes were either down modulated or unchanged (S6 Fig). SYK, JAK/STAT and β-catenin pathways were characterized by decreased p-SYK, p-STAT3 and c-JUN in TMD8^IDELA-R^. Expression of p-ERK and p-SFK remained unchanged.

### AKT and PDK1 inhibition in combination with idelalisib can overcome resistance in TMD8^IDELA-R^ cells

AKT and phosphoinositide-dependent kinase-1 (PDK1)-targeted inhibitors were used to determine if the upregulation of downstream PI3K pathway signaling was a driver of idelalisib resistance. The AKT inhibitor MK-2206 and the PDK1 inhibitor GSK2334470 had little to no single or combination agent activity on the TMD8^IDELA-S^ line (Fig 4B and 4C, left graphs). When TMD8^IDELA-R^ was evaluated for idelalisib resistance in the presence of MK-2206 or GSK2334470, sensitivity to idelalisib was partially restored relative to TMD8^IDELA-S^ (E_max_ = 60% and 59%, respectively, right graphs). As single agents, no induction of apoptosis was observed with idelalisib or GSK2334470 while a small increase was seen with MK-2206 (33%) compared to vehicle control (24%) in TMD8^IDELA-R^ (Fig 4D and 4E). Apoptosis was significantly induced with the combination of idelalisib and MK-2206 (46%) or GSK2334470 (49%) in these cells.

We next interrogated PI3K pathway modulation in the TMD8^IDELA-R^ line. As shown previously, TMD8^IDELA-R^ displayed higher constitutive activation of the PI3K pathway as demonstrated by higher levels of p-AKT S473, T308 and p-S6RP S235/236 (Fig 4F and 4G). Idelalisib alone had little to no effect on PI3K signaling in TMD8^IDELA-R^. Complete pathway suppression in TMD8^IDELA-R^ was achieved when idelalisib was combined with MK-2206 or GSK2334470, showing that PI3K pathway upregulation can be overcome by the combination of idelalisib with AKT or PDK1 inhibition.

### Profiling of ibrutinib-resistant TMD8 lines reveals BTK^C481F^ mutation and a novel A20 mutation

**Fig 5:**
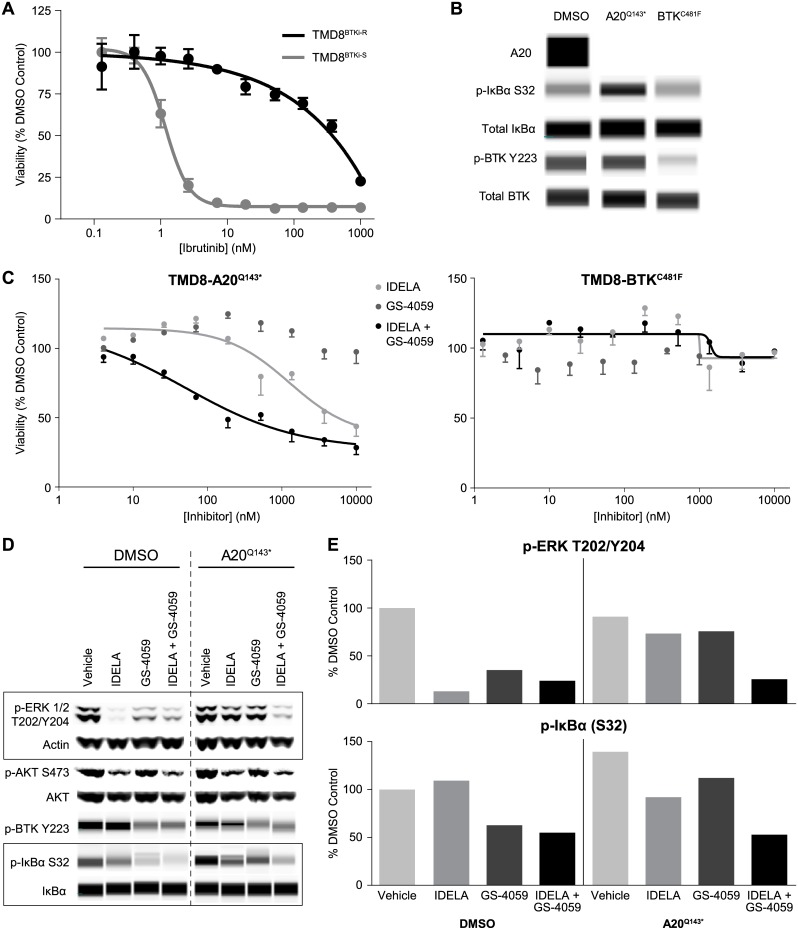
Loss of A20 and BTK C481F mutation as mechanisms of BTK inhibitor resistance in TMD8. (A) Cell viability of BTK inhibitor (BTKi)-sensitive (TMD8^BTKi-S^) and BTKi-resistant (TMD8^BTKi-R^) cells to ibrutinib, 96 hour CellTiterGlo assay, mean ± SEM, n = 4. (B) Protein lysates were generated for TMD8 (DMS0 control), TMD8^A20-Q143^*and TMD8^BTK-C481F^ cell lines, and protein expression of A20, p-IκBα, total IκBα, p-BTK and total BTK was analyzed using Simple Western. (C) Cell viability of BTKi-resistant TMD8^A20-Q143^* (left) or BTKi-resistant TMD8^BTK-C481F^ (right) treated with ONO/GS-4059, idelalisib, or a combination of idelalisib plus ONO/GS-4059 (50 nM), mean ± SEM, n = 4. (D) Lysates of TMD8 DMSO control and TMD8^A20-Q143^* lines were analyzed by western blot (p-ERK 1/2, p-AKT S473, AKT) and Simple Western (p-BTK, p-IκBα S32, IκBα). Cells were treated with idelalisib (420 nM), ONO/GS-4059 (320 nM) or in combination for 24 hours. (E) Quantification of p-ERK1/2 and p-IκBα S32 blot results from Fig 5D. Values are normalized to actin and graphed as percent of DMSO vehicle control. Simple Western and western blot images are representative experiments from n = 2–3 experiments.

We also developed a TMD8 model of acquired BTK inhibitor resistance by continuous exposure to ibrutinib in order to model the effect of idelalisib in ibrutinib-resistant disease. Two resistant lines were established by dose-escalation and continuous ibrutinib exposure at 10 or 20 nM (TMD8^A20-Q143^* and TMD8^BTK-C481F^, respectively). These lines showed complete resistance to ibrutinib and ONO/GS-4059 as compared to a passage-matched, vehicle control line (Fig 5A and 5C). WES analysis of clonal isolates from both lines revealed a homozygous mutation in BTK at C481F only in the 20 nM treated clones (TMD8^BTK-C481F^, 22/22 clones), which was confirmed by Sanger sequencing. WES analysis also revealed a novel mutation in TNFα-induced protein 3 (*TNFAIP3* Q143*mutation, A20 protein) in clonal isolates from the 10 nM treated clones only (TMD8^A20-Q143^*, 5/5 clones). Protein expression profiling in these lines showed a loss of A20 and an increase in p-IκBα in the TMD8^A20-Q143^*, signifying NF-κB pathway activation, while total and p-BTK Y223 remained unchanged (Fig 5B). A20 profiling also revealed an unexpected loss of A20 protein in TMD8^BTK-C481F^ by an unknown mechanism, as well as a decrease in p-BTK Y223.

The effect of idelalisib and ONO/GS-4059 on cell viability in the TMD8^A20-Q143^*and TMD8^BTK-C481F^ lines was then evaluated. TMD8^A20-Q143^* cells were resistant to idelalisib and ONO/GS-4059 as single agents, but some sensitivity was restored with the combination (Fig 5C, left graph). The TMD8^BTK-C481F^ line, however, was resistant to idelalisib, ONO/GS-4059, and the combination (Fig 5C, right graph), suggesting multiple and insurmountable mechanisms of resistance in this line, which includes upregulation of p-SFK, p-SYK and total SYK (S7 Fig).

Analysis of the PI3K, MAPK, BTK, and NF-κB pathway contribution to resistance was evaluated in the TMD8^A20-Q143^* line. p-ERK in the TMD8^A20-Q143^* line was insensitive to both idelalisib and ONO/GS-4059 as single agents, but was significantly inhibited with the combination of both agents (Fig 5D and 5E). This level of inhibition in the resistant line was comparable to the combination effect seen in the vehicle control line. In addition, a decrease in p-IκBα was seen with the combination of idelalisib and ONO/GS-4059, more than either single agent alone. This strong inhibition was particularly significant in light of the observed upregulation of p-IκBα, suggesting that inhibition of MAPK and NF-κB pathways might be the mechanism that leads to the decreased cell viability seen with combination treatment in this line.

## Discussion

In this study, we set out to characterize the potential antitumor activity of combining idelalisib with ONO/GS-4059, as well as to define the mechanisms of resistance for each class of agent in a model of ABC DLBCL. Our findings showed additivity at clinically relevant concentrations both *in vitro* and *in vivo*, demonstrating the effectiveness for targeting PI3Kδ and BTK in combination in this model. As expected, inhibition of both pathways suppressed downstream PI3K, BTK, and MAPK pathways in an additive manner [28–30]. Interestingly, combination treatment led to a strong inhibition of the expression of the c-MYC transcription factor, which mediates cell proliferation, apoptosis, and microenvironment remodeling [31]. It has been previously shown that idelalisib and ibrutinib can both regulate c-MYC expression in sensitive DLBCL cell lines [32]. In a recent study, ibrutinib inhibited increased transcription of c-MYC after BCR stimulation in primary CLL cells [33]. Additionally, in studies using the BET inhibitor JQ1 have shown that the ability to modulate c-MYC in models of DLBCL correlated to decreased cell viability and tumor growth suppression [34]. Collectively, the ability to suppress c-MYC is an important convergence point for both PI3Kδ and BTK inhibition in DLBCL.

Idelalisib mechanisms of resistance have previously not been characterized clinically. Our studies set out to define the mechanism of resistance at a molecular level in a model cell line. Elucidation of potential targets may provide clinical options for patients who experience disease progression. In our TMD8 idelalisib-resistance model, no mutations in *PIK3CD* or in other PI3K family isoforms were observed. While *PIK3CA* mutations and amplification, as well as an increased dependence on *PIK3CB*, have been described as resistance mechanisms in solid tumors [35–37], both our control and idelalisib-resistant lines remain insensitive to PI3Kα- and β-selective inhibitors. Additionally, our idelalisib-resistant line showed some δ-driven cross resistance to a pan-PI3K inhibitor, suggesting that a shift in dependency to other PI3K isoforms is not the driver of resistance.

While WES and protein profiling did not identify any alterations in the PI3K class I family in our idelalisib resistance model, post-translational loss of PTEN expression was observed. Loss of PTEN protein is a common mechanism observed in drug resistance and can lead to activation of downstream PI3K signaling, often in a PI3K inhibitor-insensitive manner [38]. Characterization of a breast cancer patient with resistance to the PI3Kα inhibitor BYL719 displayed altered PI3K pathway dependence due to acquisition of multiple PTEN genetic alterations in distant metastases leading to PTEN protein loss [38].

In our resistance model, bypass of the PI3Kδ requirement for PI3K pathway hyperactivation is likely due to the observed loss of PTEN. Central to this pathway is the activation of PDK1and subsequent phosphorylation and activation of AKT, which was highly upregulated in our model (16-fold). Cell survival was shown to be dependent on PDK1/AKT, as demonstrated by both cell viability and signal transduction alterations with PI3Kδ and PDK1/AKT inhibition. We also investigated whether a combination that is effective in restoring sensitivity in this resistance setting (combination of idelalisib and GSK2334470 or MK-2206) could overcome innate resistance. Of 12 DLBCL lines insensitive to idelalisib (EC_50_ > 1 μM), none displayed a shift in sensitivity to the combination of PI3Kδ and PDK1/AKT inhibition (data not shown). The same lack of ability to overcome innate resistance *in vitro* with idelalisib and ONO/GS-4059 was also observed in this cell line screen.

In parallel to the observed PI3K pathway upregulation as a mechanism of idelalisib resistance, single agent ONO/GS-4059 showed an incomplete inhibition of p-BTK and lack of effect on cell viability on TMD8^IDELA-R^. This aberrant inhibition of p-BTK, in addition to moderate effects on cell viability by targeting PI3K pathway hyperactivation, underscores previously published results which show that resistance can only be overcome by simultaneous combination inhibition leading to restoration of sensitivity to both agents [39, 40].

**Fig 6:**
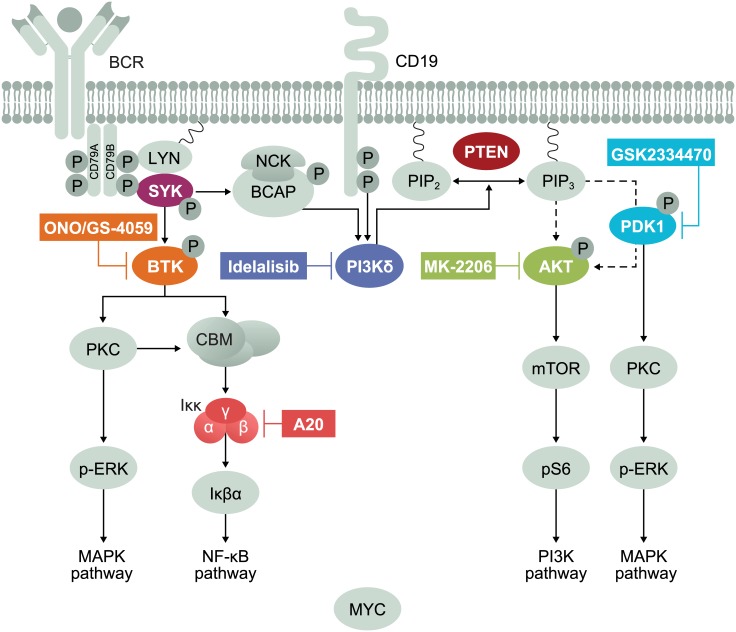
Schematic summarizing the proposed mechanisms of PI3Kδ and BTK inhibitor resistance. Upon antigen binding to the B-cell receptor (BCR), the PI3K, MAPK and NF-kB pathways are activated through a cascade of phosphorylation events. BCR stimulation recruits SYK to the membrane, where it is phosphorylated and then subsequently phosphorylates BTK. BTK activation leads to subsequent dual phosphorylation and activation of PLCγ2, which leads to downstream generation of the second messengers IP and Dag. Active PI3Kδ is recruited to the membrane and converts PIP_2_ to PIP_3_, leading to subsequent activation and phosphorylation of PDK1 and AKT. PTEN is a phosphatase which negatively regulates the PI3K pathway. PKC can be activated downstream of both PI3K and BTK leading to NF-kB and MAPK pathway activation [27]. In our idelalisib resistance model, PTEN is lost which results in the hyperactivation of the PI3K pathway. Resistance can be overcome by inhibiting PI3Kδ and the downstream phosphoproteins PDK1 and AKT. In the BTK resistance model, A20 is lost which leads to hyperactivation of the NF-κB pathway which could be overcome by inhibiting BTK and PI3Kδ. Additionally, the BTK inhibitor binding site at C481 was mutated resulting in ONO/GS-4059 resistance.

Mutations in BTK (BTK C481) and PLCγ2, have been previously associated with resistance to the BTK inhibitor ibrutinib in CLL and MCL [41–43]. Characterization of acquired BTK inhibitor resistance in TMD8 ABC DLBCL led to the discovery of a novel mutation in the *A20* gene. Our results showed that loss of *A20* expression, due to a nonsense mutation, led to upregulation of p-IκBα and loss of sensitivity to ibrutinib and ONO/GS-4059. Clinically, patients with *A20* mutations, deletions, or transcriptional down regulation did not respond to ibrutinib; conversely, ibrutinib achieved a 38% overall response rate in subjects without *A20* alterations [44]. A20, a negative regulator of NF-κB signaling, is one of the most commonly mutated NF-κB pathway genes in ABC DLBCL, which may also be a biomarker of innate resistance to therapies that target the NF-κB pathway upstream [45]. Although A20 is a negative regulator of the NF-κB pathway, it is not a direct activator. Therefore, there is likely to be an additional and unidentified driver of the NFkB pathway, downstream of PI3K and BTK. In conjunction with A20 loss, this leads to p-IκBα upregulation demonstrated by some slight resistance to PI3K or BTK inhibition alone. This points to a complex molecular mechanism leading to both PI3Kδ and BTK inhibitor resistance (Fig 6).

Upregulation of SRC family kinase (SFK) and SYK phosphorylation, in conjunction with the observed BTK-C481F mutation, suggests upregulation of an alternate survival pathway that confers cross resistance to both idelalisib and ONO/GS-4059. Targeting of the SYK pathway has been implicated recently in several reports as a mechanism to overcome drug resistance in CLL and DLBCL so it is not surprising that this may serve as a bypass mechanism of resistance [46, 47]. An additional and unexpected observation in this BTK inhibitor resistant line was a decrease in p-BTK at Y223. A recent paper has highlighted that a C481F substitution in BTK leads to catalytic inactivation and subsequent decreased autophosphorylation of BTK [48].

Clinically, there is limited experience with CLL patients in the context of disease progression while on ibrutinib or idelalisib therapy. A recent study showed that overall response rate for use of ibrutinib after idelalisib discontinuation or use of idelalisib after ibrutinib discontinuation was 77% and 50% respectively, suggesting non-overlapping mechanisms of resistance [49]. Use of combination therapy is an increasingly attractive option for patients where mechanisms of action of both drugs may act in an additive way. In particular, the effects of PI3Kδ and BTK inhibition seem to converge on common signaling pathways, thereby effectively inhibiting cell survival while utilizing lower doses of each drug. While many second generation BTK inhibitors, such as ONO/GS-4059, are in development that have better selectivity profiles than ibrutinib, our *in vitro* data demonstrate that ONO/GS-4059 is an effective BTK inhibitor and has demonstrated efficacy in the clinic [10]. Additional data using ONO/GS-4059 in combination with idelalisib shows additive anti-tumor activity *in vivo*. The evaluation of combination therapy provides an appealing mechanism to lower or limit the toxicity of each drug in a dose sparing manner. Clinical trials are currently underway to test this hypothesis with idelalisib and ONO/GS-4059 (ClinicalTrials.gov registration number: NCT02457598).

## Supporting information

S1 Fig Evaluation of PI3Kδ and BTK inhibition in OCI-LY10. Cell viability was assessed with idelalisib and ONO/GS-4059 at fixed concentrations (6, 12 or 25 nM) or with ONO/GS-4059 and idelalisib at fixed concentrations (156 or 625 nM); mean ± SEM, n = 4.
(EPS)

S2 Fig Evaluation of PI3Kδ inhibitors in TMD8. Cell viability with idelalisib and GS-649443 was assessed by CellTiterGlo after 96 hours, mean ± SEM, n = 4.
(EPS)

S3 Fig Evaluation of TMD8 clonal isolates with acquired idelalisib resistance. Clonal isolates of idelalisib resistant TMD8 and a DMSO passage-matched clone were treated with idelalisib; cell viability was assessed by CellTiterGlo assay, mean ± SEM, n = 4.
(EPS)

S4 Fig Expression of MDR genes (n = 33) in TMD8^IDELA-R^. Boxplots generated from RNAseq data, y-axis is log_2_-fold of TMD8^IDELA-S^ versus TMD8^IDELA-R^ clones, mean ± SEM.
(EPS)

S5 Fig Profiling of PI3Kγ in TMD8^IDELA-S^ and TMD8^IDELA-R^ lines. (A) PIK3CG expression levels of TMD8^IDELA-S^ and TMD8^IDELA-R^ were assessed by RNAseq analysis. (B) Protein lysates were generated from TMD8^IDELA-S^ and TMD8^IDELA-R^ cells and analyzed by Simple Western. (C) Cells were treated with the PI3Kδ/γ inhibitor IPI-145 and viability was assessed after 96 hours by CellTiterGlo assay, mean ± SEM, n = 4.
(EPS)

S6 Fig Evaluation of pathway activation in TMD8^IDELA-S^ and TMD8^IDELA-R^ lines. Protein lysates were generated for TMD8^IDELA-S^ and TMD8^IDELA-R^ cells, and analyzed by western blot (p-ERK 1/2 T202/Y204, ERK, p-STAT3 Y705, actin) or Simple Western (p-SYK Y525/526, SYK, c-JUN, p-SFK Y416, actin).
(EPS)

S7 Fig Evaluation of pathway activation in TMD8^A20-Q143^* and TMD8^BTK-C481F^ lines. Protein lysates were generated for TMD8 (DMSO control) and TMD8^BTK-C481F^ lines, and protein expression of p-SFK Y416, p-SYK Y352, total SYK and actin was analyzed by Simple Western.
(EPS)

S1 Table Activity of PI3Kδ and BTK inhibitors in DLBCL cell lines. Cell viability with ibrutinib, ONO/GS-4059, idelalisib and GS-649443 was assessed by 96 hour CellTiterGlo assay.
(PPTX)
