# Supplementary material for: PI3Kδ inhibitor idelalisib in combination with BTK inhibitor ONO/GS-4059 in diffuse large B cell lymphoma with acquired resistance to PI3Kδ and BTK inhibitors
Source: PLoS One. 2017 Feb 8;12(2):e0171221. doi: 10.1371/journal.pone.0171221 (PMC5298344; doi:10.1371/journal.pone.0171221)
Supplement: S1 Table — Cell viability with ibrutinib, ONO/GS-4059, idelalisib and GS-649443 was assessed by 96 hour CellTiterGlo assay. (PPTX) [file pone.0171221.s008.pptx]

## Slide 1
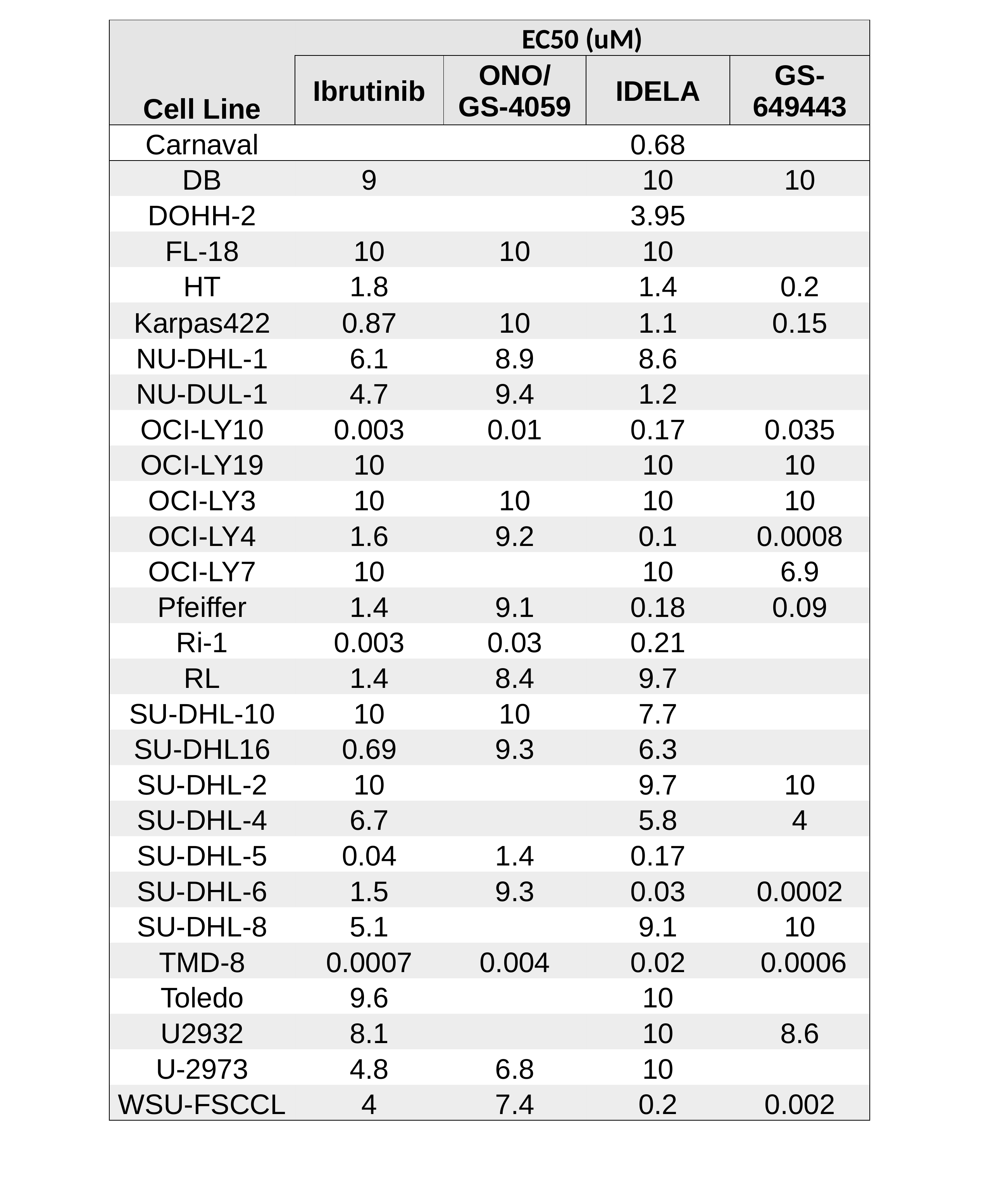

New- Supplementary Table 1
| Cell Line | EC50 (uM) | | | |
| --- | --- | --- | --- | --- |
| | Ibrutinib | ONO/ GS-4059 | IDELA | GS-649443 |
| Carnaval | | | 0.68 | |
| DB | 9 | | 10 | 10 |
| DOHH-2 | | | 3.95 | |
| FL-18 | 10 | 10 | 10 | |
| HT | 1.8 | | 1.4 | 0.2 |
| Karpas422 | 0.87 | 10 | 1.1 | 0.15 |
| NU-DHL-1 | 6.1 | 8.9 | 8.6 | |
| NU-DUL-1 | 4.7 | 9.4 | 1.2 | |
| OCI-LY10 | 0.003 | 0.01 | 0.17 | 0.035 |
| OCI-LY19 | 10 | | 10 | 10 |
| OCI-LY3 | 10 | 10 | 10 | 10 |
| OCI-LY4 | 1.6 | 9.2 | 0.1 | 0.0008 |
| OCI-LY7 | 10 | | 10 | 6.9 |
| Pfeiffer | 1.4 | 9.1 | 0.18 | 0.09 |
| Ri-1 | 0.003 | 0.03 | 0.21 | |
| RL | 1.4 | 8.4 | 9.7 | |
| SU-DHL-10 | 10 | 10 | 7.7 | |
| SU-DHL16 | 0.69 | 9.3 | 6.3 | |
| SU-DHL-2 | 10 | | 9.7 | 10 |
| SU-DHL-4 | 6.7 | | 5.8 | 4 |
| SU-DHL-5 | 0.04 | 1.4 | 0.17 | |
| SU-DHL-6 | 1.5 | 9.3 | 0.03 | 0.0002 |
| SU-DHL-8 | 5.1 | | 9.1 | 10 |
| TMD-8 | 0.0007 | 0.004 | 0.02 | 0.0006 |
| Toledo | 9.6 | | 10 | |
| U2932 | 8.1 | | 10 | 8.6 |
| U-2973 | 4.8 | 6.8 | 10 | |
| WSU-FSCCL | 4 | 7.4 | 0.2 | 0.002 |
